# A Five-Year Institutional Experience With Spatially Fractionated Radiation Therapy for Palliative Management of Bulky Tumors Across Diverse Anatomical Sites

**DOI:** 10.7759/cureus.113516

**Published:** 2026-07-28

**Authors:** Victor Caceros, Laura Ayala, Claudia Domínguez, Paola Del Cid, Aimet Siliezar

**Affiliations:** 1 Radiosurgery, International Cancer Center, Diagnostic Hospital, San Salvador, SLV; 2 Medical Unit, International Cancer Center, Diagnostic Hospital, San Salvador, SLV; 3 Medical Physics, International Cancer Center, Diagnostic Hospital, San Salvador, SLV; 4 Medicine, Universidad Dr José Matías Delgado, San Salvador, SLV

**Keywords:** bulky cancer, bulky lesion, lattice therapy, paliative care, radiotherapy (rt)

## Abstract

Introduction

Treating bulky tumors that are ineligible for surgery or resistant to chemotherapy is a significant and challenging problem. High-dose, high-precision radiation therapy is an alternative for these patients. Spatially fractionated radiation therapy (SFRT) utilizes a combination of high radiation doses targeted in an equidistant configuration and random distribution to the tumor and low-radiation doses with homogenic distribution to induce an immunological response against the tumor. The present series explores our five-year experience using SFRT as palliative treatment for bulky tumors from different anatomical locations.

Methods

From March 2019 to June 2024, we treated 21 patients with bulky tumors (>6 cm) in different anatomical locations with SFRT. Patients received doses of 18-24 Gy in high-dose regions and 5 Gy in low-dose regions. Tumoral response was evaluated through Response Evaluation Criteria in Solid Tumors (RECIST) criteria. Additionally, toxicity, changes in quality of life, and overall survival were measured.

Results

After an average follow-up of 36 weeks, 52% (n=11) patients maintained a stable disease, 38% (n=8) had a partial response, 4.76% (n=1) had a complete response, and 4.76% (n=1) had disease progression. The average tumor size at the end of treatment was 12.89 cm (SD=9.48) compared to the initial size of 15.19 cm (SD=7.48) (T-test,p=0.014). Patients had a decrease in the Visual Analogue Scale (VAS) score from 6.2 (SD=3.0) before treatment to 1.1 after (SD=1.46) (T-test,p=*0.001*). Also, patients had a global quality of life index of 0.8 at the last follow-up, with an average survival of 53 weeks. No patient presented toxicity greater than grade 2 on the Radiation Therapy Oncology Group (RTOG) scale.

Conclusions

SFRT seems to be effective for the palliative treatment of bulky tumors, as proved by the reduction of tumor volume and size, decrease of pain intensity, and improved quality of life in this series. However, randomized studies must be performed to establish the efficacy of this treatment further.

## Introduction

The palliative treatment of bulky tumors or those that have had a partial or no response to standard therapy is a very common clinical challenge. The radiosensitivity of these tumors is considered limited by their relative radio-resistance mediated by the lack of oxygen in the internal aspects of the tumor, especially in those with a high growth rate and deficient vascular neoformation [1].

It was Thomlinson and Gray [2] who initially recognized the intricate role played by low oxygen supply in the response of rapidly growing tumors to radiotherapy, demonstrating significant variations according to the hypoxic environment within them. Their conclusions contributed to the fundamental understanding of the role of tissue oxygenation in treatment response to radiotherapy. Hypofractionation schemes concomitant with chemotherapy or immunotherapy are routinely used to overcome this radioresistance. Head and neck, thorax, and pelvis tumors of epithelial histology have established and relatively successful protocols, even combined with neoadjuvant therapy [3-5].

Spatial fractionated radiotherapy (SFRT) is a method that has been used historically and has had renewed interest following several studies published since 2015. This technique is based on the application of high doses to specific selected targets distributed within the tumor in an equidistant configuration; doses are between 18 and 24 Gy Dmax and are applied in a single session on each target [6,7].

The safety and efficiency of this therapy have been improved through the use of megavoltage (MV) X-ray photons and modern techniques of dosimetry and 3D planning, such as image-guided (IGRT) intensity- modulated radiation therapy (IMRT) and intensity-modulated volumetric arc therapy (VMAT), which create marked dose gradients between high-dose and low-dose areas. Lattice radiation therapy (LRT) is a type of SFRT that uses an improved 3D technique, which has the primary objective of inducing an immunological reaction mediated by inflammation factors (RIMI) and taking advantage of well-known phenomena such as the "abscopal" and "bystander" effects to generate local and systemic control and alleviate the associated symptoms [8].

Therefore, this retrospective study aims to determine the local efficacy and safety of SFRT in patients with bulky radioresistant tumors, as well as its effect on symptom relief and quality of life in palliative patients.

## Materials and methods

Population

This multicentric study consists of a retrospective review of 60 patients treated with palliative SFRT, from March 2019 to November 2023, in two specialized centers, at the Social Security Institute of El Salvador and the International Cancer Center of El Salvador. However, control imaging was obtained only from 21 patients and quality of life monitoring was obtained in 14 of them. All patients were diagnosed with bulky tumors (>100 cc) in different anatomical locations through computed tomography (CT) and were treated with at least one session of SFRT applied with a linear accelerator with IMRT-IGRT technique from November 2018 to May 2024. Subsequently, 20 out of 21 patients were treated with conventional radiotherapy schemes with palliative intent on an average of 26 weeks after SFRT. The patients were not candidates for surgery and were being managed by either palliative medicine or algology, or a combination of the two. All patients were evaluated by the ordinary session of the radio-oncology service to proceed. Before treatment, pain intensity and symptoms were evaluated. These same parameters and toxicity were evaluated post-treatment at 10 weeks.

SFRT technique

**Figure 1:**
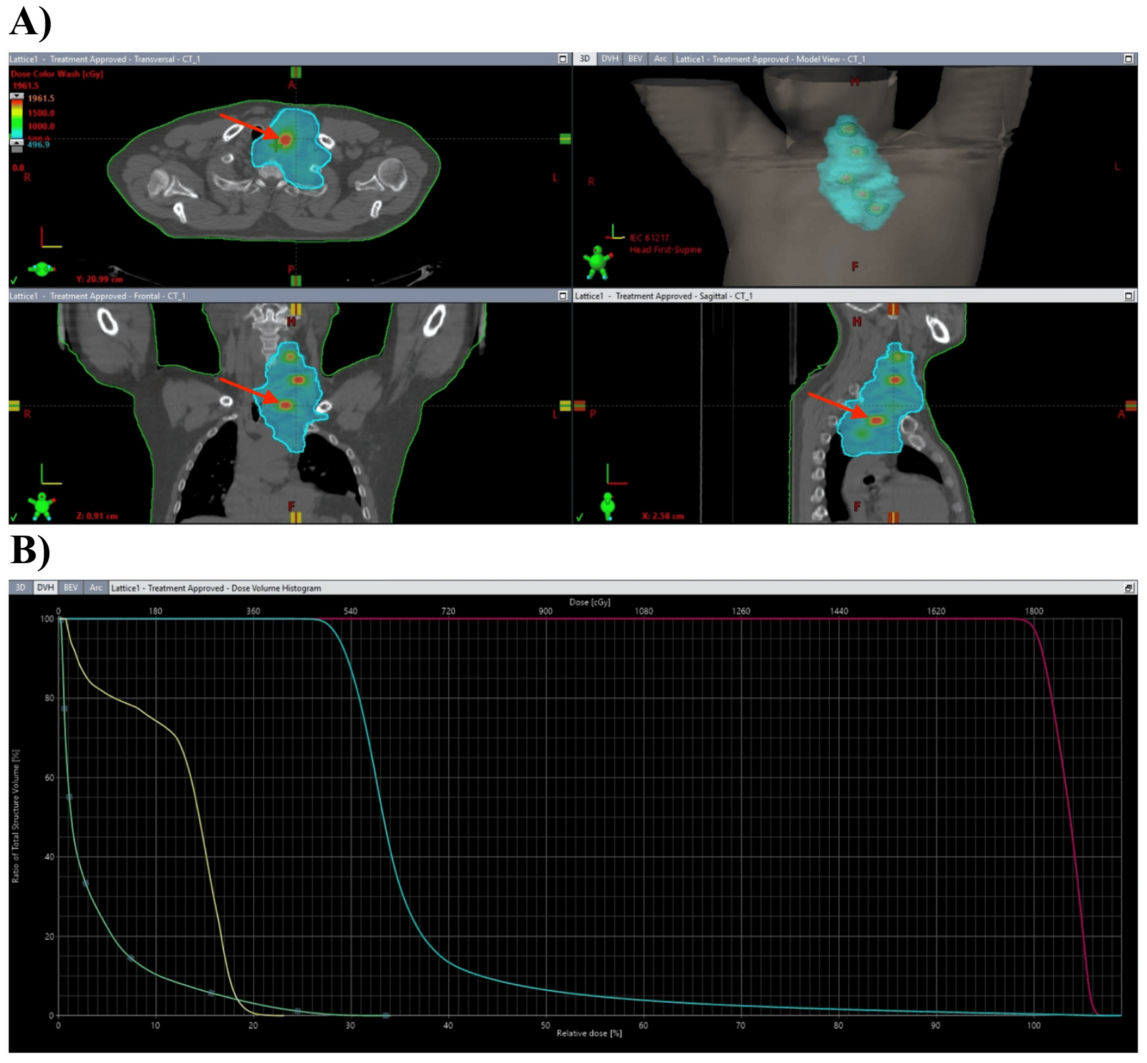
SFRT planning. A. Patient with metastatic sarcoma to the neck, treated with SFRT with doses of 18 Gy in the vertices (red arrows) and 5 Gy in the valleys (light blue shaded area). B. Volume-dose histogram: The red line represents the dose of 18 Gy at 100% to each vertex, while the aqua line represents the dose of 5 Gy at 85% in the valleys. SFRT: Spatial fractionated radiotherapy treatment.

The procedure was performed by radiation oncologists. All patients were simulated with a vacuum corporeal immobilizer, in a standard position. Simulation was performed with Tac Simulator Somatom^TM^ (General Electric, Health Care, CA. USA) using 16 slices with a thickness of 1.25-5 mm, pitch (factor that determines how fast the CT scanner table moves relative to the width of the X-ray beam during a single rotation) of 1.25-5 mm, FOV (fill of view) 20-25 cm per side, with 120-140 kV, 100-300 map using 100 ml of “Omnipaque” contrast applied with a continuous infusion pump, “Stellant” (Henry Schein R Melville, NY). The images were transferred to the ECLIPSE R dosimetric planning system provided by Varian^TM^ (Varian Medical Company, Inc., Palo Alto, CA) and applied in linear accelerator with IMRT-IGRT (Figure 1).

For SFRT, lattice technique (concept of a universal energy matrix in Jacobo Grinberg's theory) was designed in a CT scan of of 1-3 mm slice thickness that included the treated tumor lesion, along with 4-5 cm of tissue above and below. High-dose regions or “vertices” of 1-1.5 cm^3^ per 100-150 cm^3^ of tumor volume (GTV) were targeted in an equidistant manner across different planes, at a distance of 4 cm between each other and more than 2 cm from the outer edge of the tumor; the prescribing dose was 18-24 Gy in the vertices and 5 Gy to the valleys or total tumor volume (Table 1). This was determined by a team of senior radiation oncologists and physicists, according to the dosimetry analysis, the complexity of the GTV, and its proximity to deep-seated organs at risk (OARs) and skin. Tumor response was evaluated eight to 10 weeks later through a comparative CT scan with a thickness of 3-5 mm.

**Table 1:** Dosification characteristics of SFRT. SFRT: Spatially fractionated radiation therapy; GTV: gross tumor volume.

Patient	GTV	Dose GTV	Number of vertices	Volume of vertices	Dose vertices
1	3397.93	7.06	12	7.52	18.54
2	740.42	8.98	6	14.2	18.62
3	359.49	6.48	5	1.73	18.64
4	231.54	7.97	5	7.17	19.03
5	110.92	6.9	1	1.65	18.75
6	3775.5	2.4	12	15.54	20
7	533	6.54	8	2.77	19.08
8	178.64	7.23	2	1.25	21.04
9	6029.08	7.68	7	27.19	18.78
10	289.9	6.98	4	1.38	18.6
11	1583.75	4.4	13	10.53	18.95
12	145.7	3.2	1	1.48	20.83
13	383.5	2.1	10	14.64	20
14	475.57	6.21	3	4.52	19
15	516.84	7.3	4	5.53	18.47
16	125.9	6.94	2	3.29	18.76
17	2427.6	5	22	75.56	18
18	2153.8	5	21	31.03	18
19	760.52	4.7	8	30.39	18
20	1633.9	7.6	11	17.8	19.25
21	119.6	7.22	2	2.1	19.06

RECIST criteria

Treatment results were evaluated using the Response Evaluation Criteria in Solid Tumors (RECIST) version 1.1 criteria [9]. Baseline CT imaging was performed at a maximum of one month prior to treatment. The target lesion was chosen, and the longest diameter was measured. Only in one patient were two targets selected, for which the sum of the length (SLD) of both targets was calculated. At an average of 10 weeks post-treatment, control CT was performed to evaluate tumor response. Patients were classified into four groups, according to the longest diameter measured: (i) complete response (CR), disappearance of all target lesions; (ii) partial response (PR), decrease of 30% or more in length diameter or SLD; (iii) stable disease (SE), no PR or DP; and (iv) disease progression (DP), increase of 20% or more in length diameter or SLD.

Toxicity and quality of life 

Through follow-ups, acute adverse effects were recorded using the toxicity criteria of the Radiation Therapy Oncology Group scale (RTOG), according to the affected organs and which were measured on a scale of 1 to 5 according to their severity. Pain intensity was evaluated using the Visual Analogue Scale (VAS), which gives a numerical value from 0 to 10. Finally, quality of life was measured using the EuroQol 5-Dimensions 5-Levels (EQ-5D-5L) scale [10], which includes the dimensions of mobility, self-care, usual activities, pain/discomfort and anxiety/depression, giving them a score from 1 to 5, with which a global score is subsequently obtained. Additionally, the Karnofsky Performance Status (KPS) [11] was measured before treatment.

## Results

The study population consisted of 47.2% women and 52.4% men, with a mean age of 50.8 years (range: 24-77 years). All patients had bulky tumors, with an average length of 15.2 cm (range: 6.78-27.49 cm) and an initial mean tumor volume of 1236.8 cm³ (range: 110.92-6029.08 cm³). Sarcomas were the most common tumor type, observed in seven patients, followed by squamous cell carcinoma in six patients, while one patient was diagnosed with a mixed non-seminomatous germ cell tumor. Additionally, 13 patients experienced oncologic pain, with a median pre-treatment VAS score of 7. The KPS scores ranged from 50 to 80, with a median score of 70 (Table 2).

**Table 2:** Demographic characteristics. SFRT: Spatial fractionated radiotherapy treatment; F: Female, M: male.

Variable	Value
Gender, n (%)	
F	10 (47.6%)
M	11 (52.4%)
Age	50.8 (24-77)
Primary tumor type, n (%)	
Sarcoma	7 (33.3%)
Squamous carcinoma	6 (28.6%)
Adenocarcinoma (prostate and endometrial)	2 (9.5%)
Mixed non-seminomatous germ cell tumor	1 (4.8%)
Cancer of unknown primary	5 (23.8%)
Pre-SFRT tumor size (cm), mean (SD)	15.5 (7.48)
Pre-SFRT tumor volume (cm³), mean (SD)	1236.8 (1551.6)
Pre-SFRT VAS median (range)	7 (0-10)
Pre-SFRT KPS, median (range)	70 (50-80)
Follow up in months, mean (range)	13.25 (1-52)

Tumor response 

**Figure 2:**
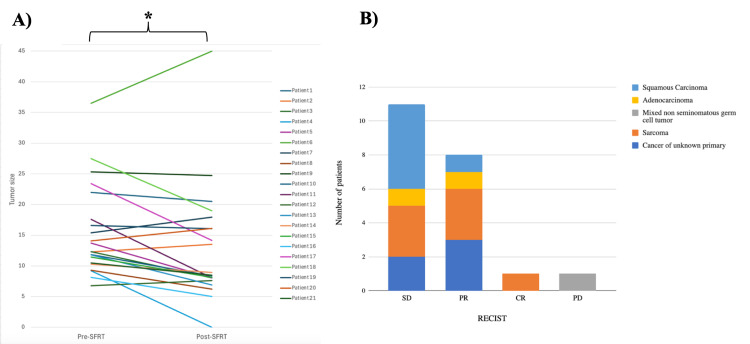
Tumor response after treatment. A. Tumor size before and after SFRT treatment (cm). B. Classification of patients according to tumor histology and tumor response after treatment. SFRT: Spatial fractionated radiotherapy; RECIST: Response Evaluation Criteria in Solid Tumors; pre-SFRT: before SFRT; post-SFRT: last follow-up after SFRT.

A response to treatment was observed in 95.2% (n=20) of patients within an average of 18 weeks. At last follow-up, one patient, diagnosed with mixed mesenteric liposarcoma and an initial tumor length of 9.2 cm, achieved a complete response. Nineteen patients (36.4%) exhibited either a partial response or stable disease, while only one patient experienced disease progression. A statistically significant association was found between treatment response and tumor histological type (chi-square, p=0.012) (Figure 2). Patients with sarcoma and squamous carcinoma responded better to treatment. Both histological types showed clinical benefits, presented with stable disease, and showed a partial response. Furthermore, one patient with sarcoma presented with complete response. On the contrary, just one patient with mixed non-seminomatous germ cell tumor presented with progression of disease.

The mean tumor size significantly decreased from 15.5 cm (range: 6.78-36.48 cm) before treatment to 12.8 cm (range: 0-45 cm) after treatment (T-test, p=0.014). Similarly, the mean tumor volume decreased from 1236.8 cm³ (range: 110.9-6029 cm³) to 870.6 cm³ (range: 0-5373 cm³), although this change did not reach statistical significance (Wilcoxon W, p=0.277).

**Figure 3:**
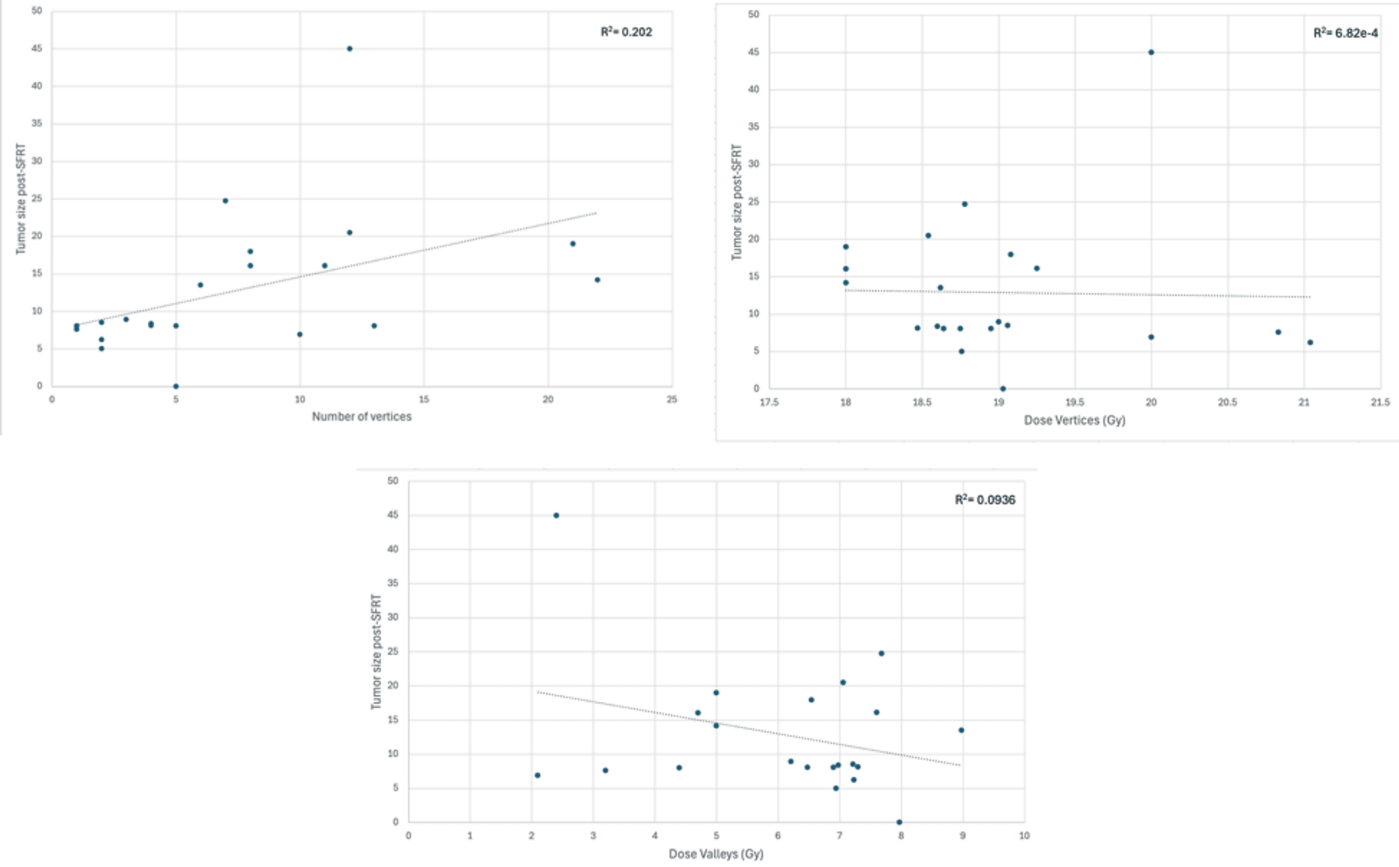
Association between tumor size after treatment and the number of vertices applied, dose applied in vertices, and dose applied in total tumor volume. Tumor size is measured in cm, tumor volume in cm³. Post-SFRT: Last follow-up after spatial fractionated radiotherapy.

The relationship between treatment variables and treatment response in tumor size at last follow-up was analyzed (Figure 3). A statistically significant relationship was found between tumor size after treatment and the number of vertices applied (Spearman’s rho, R2=0.202, p=0.007). Alternatively, it was found that the tumor size after treatment does not show a statistically significant relationship with the dose administered in vertices (Spearman’s rho, R2= 6.82e-4, p=0.206) and valleys (Spearman’s rho, R2= 0.0936, p=0.955).

Toxicity and quality of life

**Figure 4:**
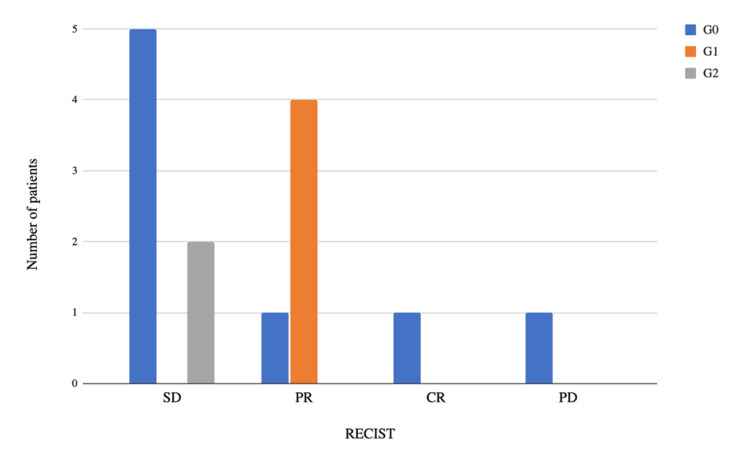
Toxicity according to tumor response. Number of patients (n) in each tumor response group presenting side effects classified as grade 0, 1, and 2 toxicity according to the RTOG toxicity criteria scale. SD: Stable disease, PD: progressive disease, CR: complete response, PR: partial response. RTOG: Radiation Therapy Oncology Group.

Information on the side-effects was obtained only from 14 patients; four patients presented grade 1 toxicity, and three of them had grade 2 according to the RTOG scale. The most common side effects were fatigue, loss of appetite, nausea, vomiting, and pain. A patient with lung metastasis presented with mild dyspnea (Figure 4).

**Figure 5:**
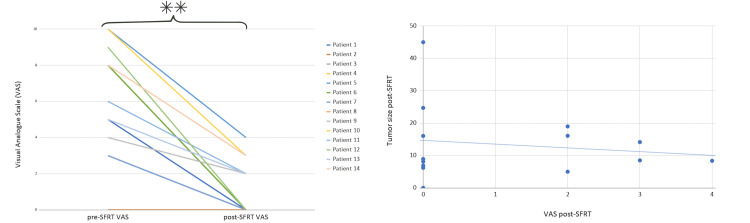
Pain intensity and tumor size after SFRT. A. Pain intensity on the Visual Analogue Scale shows a significant reduction following spatial fractionated radiotherapy (SFRT) (T-test, p=0.001). B. Pain intensity according to Visual Analogue Scale and tumor size (cm) after SFRT (Pearson test, R2=0.0231, p=0.604). Pre-SFRT: Before SFRT; post-SFRT: last follow-up after SFRT.

Patients showed a significant improvement in pain levels after SFRT, going from a VAS score of 6.2 (SD=3.0) points before SFRT to 1.1 (SD=1.46) points after treatment (T-test, p=0.001) (Figure 5). Notably, no correlation was found between post-treatment VAS and tumor size (Pearson test, R2=0.0231 p=0.604).

**Figure 6:**
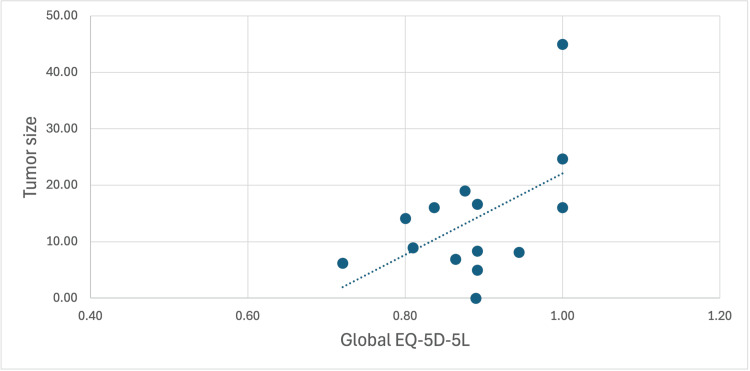
Patients' global quality of life after Spatial Fractionated Radiotherapy Tumor size is measured in cm. Global EQ-5D-5L: Global EuroQol five-dimensions five-level scale [10].

Regarding quality of life, a mean global EQ-5D-5L score of 0.84 was obtained after treatment. No correlation was found between quality of life and the size of the tumor response (Spearman’s rho, R2= 0.249, p=0.300) (Figure 6).

## Discussion

LRT-SFRT was safely administered for palliative purposes to 21 patients with voluminous tumors of different histological tumor types, resulting in the stabilization of the disease and a statistically significant decrease in tumor size. This was achieved without increasing patient morbidity or mortality; in fact, it improved their quality of life and reduced pain levels.

Retrospective studies have demonstrated the effectiveness of SFRT in treating bulky, radioresistant tumors for various therapeutic purposes [12]. One key application is reducing tumor mass to facilitate subsequent chemotherapy or radiotherapy, improving the response to these treatments [13, 14]. Additionally, SFRT can serve palliative purposes, providing rapid symptom relief [15], as exemplified by the findings in the present study.

Our results are consistent with previous reports in which a reduction in tumor size was demonstrated in 53% of patients and a complete response in 55%, without causing significant side effects or decreasing survival [16, 17]. In contrast to these studies, our sample includes a diverse range of lesion types, and a different treatment regimen was applied; nevertheless, we achieved similarly positive responses.

Also, we observed in our study that tumors with a histology of carcinoma and sarcomas responded better to treatment. Similar responses were observed in a cohort of 81 tumors, where >70% of cases responded to treatment and the mean extent of shrinkage in carcinomas and sarcomas was similar (~50% of initial volume) but higher compared to melanoma (~28%) [18]. However, studies with a larger sample are needed to determine a statistical relationship in these findings.

LRT involves establishing a high-low dose distribution by placing multiple high-dose vertices within the tumor volume using a 3D conformational IMRT technique [19]. The layout recommendations are to create high-dose vertices of 0.5-1.5 cm in diameter distributed within the gross tumor volume with a 2.0-5.0 separation from each other, and with an inward margin of 1-2 cm from the tumor boundary to avoid excessive dose to the surrounding normal tissue. The dose in the valleys should be low enough to prevent tumor destruction, with a recommendation of keeping it below 5 Gy; and the dose in the vertices can vary depending on treatment objectives, for palliative or boost treatment, it can range from 5-20 Gy and for induction of anti-tumor immunity is from 15-25 Gy [20-22].

Our study treatment planning parameters were in accordance with these recommendations. The results demonstrated that tumor reduction and symptomatic improvement - particularly in pain relief and quality of life - were more pronounced in patients who received doses close to 18 Gy in the vertices and 5 Gy to the GTV. Additionally, a target distribution of one vertex per 100-150 cc of tumor tissue appeared optimal for achieving these results. This distribution ensures a logical and spatially uniform arrangement, while promoting an appropriate dose-gradient distribution between high-dose and low-dose regions. This spatial configuration is considered critical for the induction of the bystander effect within the tumor microenvironment previously explained. Future comparative randomized studies should explore whether increasing the number of targets or altering their spatial distribution influences treatment outcomes.

This clinical efficacy can be explained by the underlying biological mechanism of LRT that extends beyond direct radiation-induced cytotoxicity through the induction of the bystander effect. The high-dose vertices induce DNA damage and immunogenic cell death. These promote the release of damage-associated molecular patterns, cytokines, and reactive oxygen species, which stimulate the recruitment of lymphocytes to initiate an adaptive antitumor immune response and immunogenic signals. These signals diffuse to the low-dose valleys, triggering immune activation. Also, low-dose irradiation remodels the tumor microenvironment by promoting vascular and stromal normalization, decreases the expression of immunosuppressive mediators, and increases the secretion of pro-inflammatory chemokines. Together, these changes amplify tumor cell killing beyond the irradiated vertices while facilitating immune-cell infiltration and antigen presentation [18,23].

Studies have shown that LRT can also induce an abscopal effect, which represents a systemic immune response in which localized irradiation results in regression of metastatic lesions. Within the high-dose vertices, immunogenic cell death promotes the release of tumor neoantigens that are captured by dendritic cells and presented to naive T cells in tumor-draining lymph nodes, generating tumor-specific cytotoxic CD8+ T lymphocytes. These T cells circulate systemically, while recognizing and eliminating metastatic tumor cells [24]. Although this phenomenon remains relatively uncommon with radiotherapy alone, accumulating preclinical and emerging clinical evidence suggests that LRT may potentiate the efficacy of immune checkpoint inhibitors [25] . Notwithstanding, previous studies have demonstrated bystander and abscopal response rates of 96% and 52%, respectively, obtaining a partial response in bulky tumor and non-irradiated metastases [26,27].

Currently, there is limited evidence about the effect on the patient's quality of life after presenting stabilization or complete response to the disease; however, minimal adverse effects, relief of symptoms, and prolongation of survival have been observed [28,29]. In a study by Ahmed et al., 60% of patients had symptom relief 1.6 months after treatment. In addition, there was an overall survival rate of 53% per year. Regarding toxicity, 20% presented grade 1 and 2 toxicity, and 8% presented grade 3 and 4 [30].

In our study, all patients except one reported over 76% of pain relief, the patient had a mean survival of 13.25 months, with 46% experiencing grade 1 or 2 toxicity. Following treatment, the mean global quality of life score (EQ-5D-5L) was 0.84. Regarding quality of life, before treatment, patients exhibited KPS lower than 80 points. After treatment, all patients reported a score greater than 80. Also, a mean global score (EQ-5D-5L) of 0.84 was obtained after treatment. No correlation was found between KPS or EQ-5D-5L score and the size of the tumor response (Spearman’s rho, R2= 0.312, *p=0.277/Spearman’s rho, R2= 0.249, *p=0.300, respectively) (Figure 6). Notably, quality of life, pain relief, and overall survival appeared to be significantly associated with tumor response, highlighting the potential impact of treatment outcomes on patient well-being.

Our findings suggest that rapidly growing epithelial tumors may elicit a stronger immune response, contributing to favorable treatment outcomes, as evidenced by radiological improvements and enhanced quality of life. However, an equivalent response rate was not consistently observed, even among tumors with similar histology and dimensions. This variability may be attributed to differences in individual immune responses, which are influenced by factors such as nutritional status, comorbidities, and concurrent medications. These variables complicate the measurement and achievement of the abscopal effect, underscoring the need for further investigation in randomized studies.

The important limitations of the study are the small sample size and the limited follow-up, which was due to the few patients who met the study criteria and the patients who were lost during imaging and clinical follow-up. Furthermore, this study did not evaluate the systemic effect of the treatment nor the association of the different percentages of tissue oxygen concentration in necrotic, hypoxic, or adequately oxygenated areas and their response to SFRT. It is necessary to carry out randomized studies to corroborate these initial results and to establish an association between the treatment response and the factors that may influence it.

## Conclusions

This retrospective study supports that SFRT is both effective and safe for treating bulky tumors, particularly those of epithelial origin, with a palliative intent. Besides tumor shrinkage, this treatment relieves symptoms and improves quality of life. The magnitude of the observed clinical benefit is particularly relevant in patients who have exhausted multiple therapeutic options and whose quality of life has been profoundly affected by pain and disease burden. Nevertheless, it remains premature to consider this strategy as a curative modality or as a substitute for established standard-of-care treatments, such as surgical resection when technically feasible and clinically indicated. In addition, the complex role of the immune system, including the impact of immune competence on therapeutic response and the mechanisms through which these responses may be enhanced, is not yet fully understood. Future translational and clinical research should explore the effects of this approach across various tumor types, investigate combinations with other treatment techniques, and elucidate the biological mechanisms and variables that contribute to treatment outcomes.
